# Dielectrophoretic Microfluidic Device for in Vitro Fertilization

**DOI:** 10.3390/mi9030135

**Published:** 2018-03-20

**Authors:** Hong-Yuan Huang, Yun-Li Lai, Da-Jeng Yao

**Affiliations:** 1 Department of Obstetrics and Gynecology, Chang Gung Memorial Hospital, 5, Fu-Hsing Street, Kwei-Shan, Tao-Yuan 333, Taiwan; hongyuan@cgmh.org.tw; 2 Department of Obstetrics and Gynecology, Chang Gung University and College of Medicine, 259, Wen-Hua 1st Road, Kwei-Shan, Tao-Yuan 333, Taiwan; 3 Department of Power Mechanical Engineering, National Tsing Hua University, 101, Section 2, Kuang-Fu Road, Hsinchu 30013, Taiwan; s7537877@hotmail.com.tw; 4 Institute of NanoEngineering and MicroSystems, National Tsing Hua University, 101, Section 2, Kuang-Fu Road, Hsinchu 30013, Taiwan

**Keywords:** microfluidics, dielectrophoretic, insemination, in vitro fertilization

## Abstract

The aim of this work was to create a microfluidic platform that uses in vitro fertilization (IVF) and avoids unnecessary damage to oocytes due to the dielectrophoretic force manipulation of the sperms and oocytes that occurs in a traditional IVF operation. The device from this research can serve also to decrease medium volumes, as well as the cost of cell culture under evaporation, and to prevent unnecessary risk in intracytoplasmic sperm injection (ICSI). To decrease the impact and destruction of the oocyte and the sperm, we adopted a positive dielectrophoretic force to manipulate both the sperms and the oocyte. The mouse oocytes were trapped with a positive dielectrophoretic (p-DEP) force by using Indium Tin Oxide (ITO)-glass electrodes; the ITO-glass electrode chip was fabricated by wet etching the ITO-glass. The polydimethylsiloxane (PDMS) flow-focusing microfluidic device was used to generate microdroplets of micrometer size to contain the zygotes. The volume of the microdroplets was controlled by adjusting the flow rates of both inlets for oil and the DEP buffer. As a result, the rate of fertilization was increased by about 5% beyond that of the DEP treatment in traditional IVF, and more than 20% developed to the blastocyst stage with a low sperm-oocyte ratio.

## 1. Introduction

In vitro fertilization (IVF) is one of the most effective treatments for infertility, commonly applied when other methods of achieving conception have failed. With the increasing clinical utilization of assisted reproductive technology (ART), scientists and clinicians have acquired insight into the basic biology of gametes and embryos and have translated that knowledge into improved rates of success following assisted reproduction. Over the past three to four decades, significant improvements in human ART outcomes have been realized. Laboratory techniques, equipment, media, and their collective environments have altered over time and have positively influenced each step in human IVF, embryo culture and analysis, and embryo cryopreservation processes. These approaches aimed to improve embryo development in vitro have involved mostly the chemical composition of the culture media. Both conventional and sequential culture-medium systems have been refined; the development of high-quality blastocysts in vitro is common in clinical practice with a good prognosis [1,2,3].

IVF is a technique in which sperms and oocytes are conventionally inseminated and cultured in specialized laboratory conditions. The introduction of intracytoplasmic sperm injection (ICSI) revolutionized the treatment of couples with male factor infertility and enabled paternity for a large proportion of men with nonobstructive azoospermia, or no measurable sperm count [4,5]. The use of ICSI for patients with borderline or even normal semen characteristics has increased predominantly [6], with no clear evidence of benefits of using ICSI over conventional in vitro fertilization [7,8,9].

In contrast to conventional IVF, ICSI bypasses natural barriers to fertilization, thereby increasing the possibility of the transmission of genetic defects from one generation to the next. Pregnancies resulting from the application of ICSI have been associated with up to four times increased incidence of chromosomal abnormalities, imprinting disorders, autism, intellectual disabilities, and birth defects [10,11,12,13,14,15] compared with pregnancies deriving from conventional IVF. These increased risks might be related either to the effects of underlying male or female sub-fertility, or to other medical factors present in couples who are candidates for ICSI, or to the ICSI procedure.

ICSI is a technique in which single sperm are injected directly into the oocyte for fertilization; it might cause unexpected damage due to manipulation of the sperms and oocyte [16]. Only recently have biomimetic and microfluidics become an approach to increase the rate of success of IVF. Additional implemental and analytical approaches and techniques have been reported; an examination of various novel culture platforms to explore the impact of physical and mechanical modifications on the embryo might emerge in improving further the development in vitro [17,18,19,20].

Several novel culture techniques have been developed for the help of embryo culture, such as oocyte microenvironment pretreatment, sperm sorting, and media volume adjust [18,20,21], but the manipulation of multiple oocytes in a continuous fluidic channel must still be overcome to prevent the contamination of bio-reactions. Oocytes subjected to an electric treatment had a developmental potential better than those without treatment [22]. The rate of fertility was improved with smaller concentrations of sperms using a microfluidic device [23]. The aim of this work was to develop a lab-on-a-chip (LOC) system for the control of insemination by sperms and oocyte. This technique might provide benefit for an oligozoospermia patient to achieve a high rate of fertilization with IVF and a limited number of oocytes as well as a small concentration of sperms. We therefore used a droplet-based biochip to transport, sort, and store the oocyte, sperms, and embryo using a dielectrophoretic (DEP) effect and a microfluidic system.

## 2. Materials and Methods

### 2.1. Gamete Collection

The experiments were approved by the Chang Gung Memorial Hospital (CGMH) Animal Care and Use Committee and conducted in accordance with the principles and procedures outlined in the CGMH Guidelines for the Institutional Animal Care and Use Committee (IACUC #2010083002). The oocytes were obtained from six-week-old Institute of Cancer Research (ICR) mice as reported previously [24]. Briefly, the females were superovulated with an intraperitoneal injection of pregnant mare-serum gonadotropin (PMSG, 5IU) to stimulate the development of ovarian follicles, followed by human chorionic gonadotropin (hCG, 5IU) 42–48 h later. After hCG administration, the superovulated mice were euthanized; the oocytes were obtained on flushing the oviducts 10–13 h later. The oocytes were pre-treated with a micropipette and cultured in a Potassium Simplex Optimized Medium (KSOM) medium. The males were euthanized with cranial/cervical dislocation. The cauda portion of the epididymis was minced with scissors and added to the medium. After allowing sperms to swim for 1 h, the sperms for insemination were collected and incubated at 37 °C in an atmosphere of CO_2_ (5%) in air. The concentration of sperms in all experiments was about 1.5 × 10^6^ sperm/mL.

### 2.2. Theory

The calculation of the dielectrophoretic force for spherical particles is based on:

(1) $$\vec{F}=2\pi r^{3}\varepsilon_{m}Re\left[CM\left(\omega \right)\right]\nabla {\vec{E}}_{rms}^{2}$$

in which *r* is the radius of the particle, $\varepsilon_{m}$ is the dielectric permittivity of the medium, $\nabla {\vec{E}}_{rms}^{2}$ is the root-mean-square value of the electric field, and Re[CM(ω)] is the real part of the Clausius-Mossotti factor. This Clausius-Mossotti factor is typically in the range of −0.5 to 1 and is defined as follows:

(2) $$CM\left(\omega \right)=\frac{\varepsilon_{p}^{*}-\varepsilon_{m}^{*}}{\varepsilon_{p}^{*}+2\varepsilon_{m}^{*}}$$

ε is the dielectric permittivity of a particle (subscript *p*) and medium (subscript *m*). Each complex form refers to $\varepsilon^{*}=\varepsilon -j\left(\sigma /\omega \right)$ in which $j=\sqrt{-1}$ and σ is the conductivity of the medium and particle. If Re[CM(ω)] > 0, the particle becomes pushed towards the regions of a strong electric field, called a positive dielectrophoretic response (p-DEP). On the contrary, if Re[CM(ω)] < 0, the particle becomes repelled from the region of strong electric field, called a negative dielectrophoretic response (n-DEP). In this work, we predicted the dielectrophoretic phenomenon of the oocytes and sperms from the research of Jones et al. [25], who presented the Protoplast Model to modify CM(ω) as follows:

(3) $$CM\left(\omega \right)=-\frac{\omega^{2}\left(\tau_{1}\tau_{m}-\tau_{c}\tau_{m}^{*}\right)+j\omega \left(\tau_{m}^{*}-\tau_{1}-\tau_{m}\right)-1}{\omega^{2}\left(2\tau_{1}\tau_{m}+\tau_{c}\tau_{m}^{*}\right)-j\omega \left(\tau_{m}^{*}+2\tau_{1}-\tau_{m}\right)-2}$$

τ=ε/σ, $\tau_{m}=\left(C_{mb}R\right)/\sigma_{c}$, and $\tau_{m}^{*}=\left(C_{mb}R\right)/\sigma_{l}$ are dimensionless constants, subscript *c* represents cell, subscript *l* represents liquid, $C_{mb}$ is the conductance of the cell membrane, *R* is the radius of a cell, σ is the conductivity, and ε is the permittivity. In this work, we predicted the DEP phenomena of the oocytes and sperms with this protoplast model.

### 2.3. Chip Design

**Figure 1:**
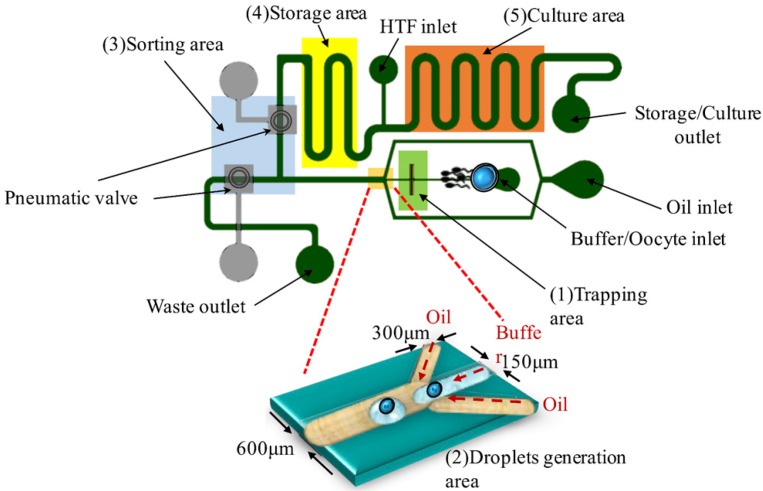
Design of the droplet-based dielectrophoretic (DEP) microfluidic biochip. The height of the channel is about 140 µm to let the oocytes (80 µm) pass into the channel.

**Figure 2:**
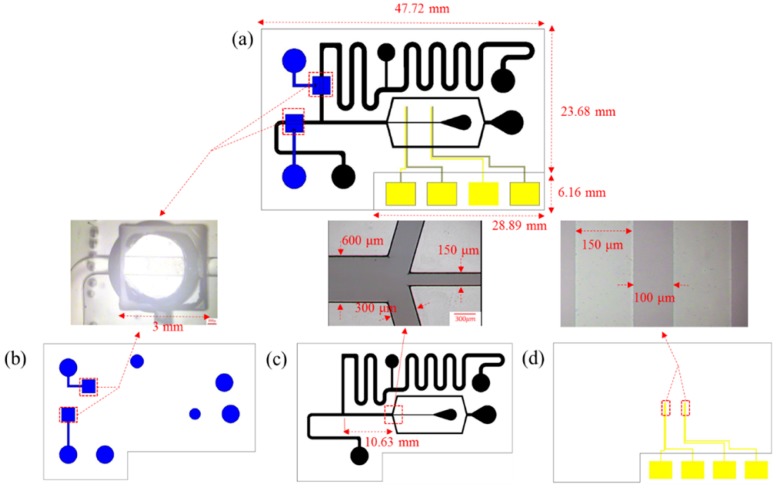
Sizes of all parts in the chip. The total chip size is 47.72 mm × 29.84 mm. We left an area of 28.89 mm × 6.16 mm for the ITO pads to connect to a function generator.

The design of a microfluidic device is separable into four sections, as shown in Figure 1. (1) Trapping area: a positive DEP force is used to manipulate the oocyte and to concentrate the sperms on the Indium Tin Oxide (ITO) electrodes; (2) Droplet generation: a flow-focusing design is used to generate emulsion microdroplets within separate oocytes (volume about 100 nL); (3) Sorting area: a pneumatic valve is used to sort microdroplets with fertilized zygotes; (4) Storage area: microdroplets are stored and cultured. The size of the chip is 47.72 mm × 29.84 mm, as shown in Figure 2.

### 2.4. Chip Fabrication

**Figure 3:**
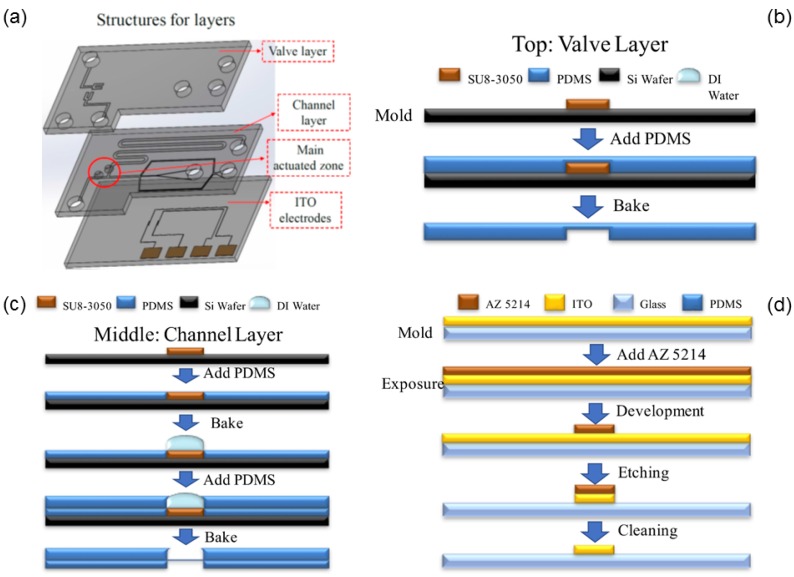
Fabrication of the DEP microfluidic in vitro fertilization (IVF) biochip. (**a**) Concept of the chip design in a three-dimensional (3D) condition. Following are the fabrication of the (**b**) top layer, (**c**) middle layer, and (**d**) bottom layer. The structure mold of the microchannel is fabricated with SU8 soft lithography on a silicon wafer with polydimethylsiloxane (PDMS) to copy the mold. The PDMS structure was bonded to the ITO electrode chip with oxygen plasma.

The fabrication of this microfluidic IVF biochip is separable into three parts, as shown in Figure 3a. The first part is the top layer that contains space for air pressure. Following is the middle layer that contains the valve membrane and the channel. The bottom layer is the ITO-glass electrode. The three parts are bonded with an oxygen plasma treatment.

● Top layer

A soft lithography technique was used to fabricate the mold in this research. The negative photoresist (SU8-3050) was coated at two spin speeds, 500 rpm for 10 s and 850 rpm for 30 s. The total uniform thickness of the SU8 3050 mold was about 140 µm. To slowly remove the solvent in the photoresist, the soft baking parameter was set at 5 °C per 3 min to 65 °C (heating 30 min) and 95 °C (heating 1 h). After UV exposure, SU8 developer was used to develop the photoresist. When the mold was completed, we poured the liquid polydimethylsiloxane (PDMS) (A:B = 10:1) into the microstructure mold and removed the bubbles with a vacuum pump. We then baked the PDMS for 30 min at 85 °C to solidify the PDMS. We finally separated the PDMS from the mold. The fabrication process is shown in Figure 3b.

● Middle layer

The soft lithography technique and PDMS were used to fabricate this layer, using a different method to form the valve membrane area. We poured the PDMS into the mold and spun it at 3000 rpm for 30 s, and then baked it at 85 °C for 15 min for the first time. After that, we added two deionized water droplets (15 µL) on a particular area per chip and poured PDMS (15 g) for a second baking at 85 °C. The water droplet was dipped on the surface physically to create the membrane structure while gently pouring the second PDMS layer. The second PDMS layer started curing before the evaporation of the droplet during baking. We removed the residual water on the PDMS layer after baking for 20 min to complete the fabrication of this layer. The process is shown in Figure 3c.

● Bottom layer

We first cleaned the ITO-glass substrate with acetone, isopropanol (IPA), and deionized water. hexamethyldisilazane (HMDS) vapor was then deposited on the substrate for 5–10 min. The positive photoresist (AZ5214) was spin-coated at 3000 rpm for 30 s. There followed soft baking at 100 °C for 1 min and UV exposure. AZ 400 K was used to develop the photoresist (AZ 400 K:DI water = 1:5). After the electrodes were patterned, we etched the ITO-glass substrate with diluted aqua regia solution (DI water:nitric acid:hydrochloric acid = 1:0.08:1) at 45 °C for 150 s. We removed the residual positive photoresist with ALEG-310 at 60 °C for 5 min to complete the ITO-glass electrode chip. The process is shown in Figure 3d.

● Bonding

The PDMS microfluidic-microstructures and ITO-glass electrodes chip were bonded to each other from top to bottom with oxygen plasma [26] to complete the production of the DEP microfluidic IVF biochip. The next step was to heat the chip on a hotplate at 85 °C for 20 min to increase the bonding force. Finally, we punched a hole (diameter 3.0 mm) as the inlet reservoir.

### 2.5. Experimental Process

In this research, we separated the samples into two groups for DEP treatment and traditional IVF control. We particularly focused on a small sperm-oocyte ratio (the distribution of sperms per oocyte) for fertility to imitate the real situation of an oligozoospermia patient.

● Standard IVF control group

In this research, we compared the in vitro fertilization rate with that in the DEP treatment experiment. The sperm-oocyte ratio represents the total number of sperms for each oocyte. We set the sperm-oocyte ratio as 500 to 25,000; the sample was incubated in a humidified incubator (5% CO_2_ at 37 °C).

**Figure 4:**
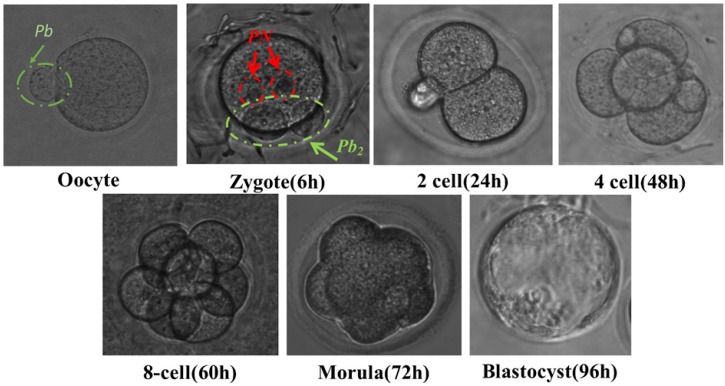
Stage of embryo development at varied intervals. The second polar body (Pb_2_) and two pronuclei (PN) were observed about 6 h after fertilization.

We prepared droplets (8 μL) for 20 sets at a sperm-oocyte ratio of 500 to 25,000. The sperms and oocytes were co-incubated for 1 h in a humidified incubator (5% CO_2_ at 37 °C). After insemination, all oocytes of these sets were washed three times in a KSOM-AA medium; each oocyte was transferred into another fresh pre-equilibrated KSOM-AA droplet (8 µL) for 20 sets covered with mineral oil in a Petri dish. After that, we incubated the dish in a humidified incubator (5% CO_2_ at 37 °C). The embryo development was then tracked and observed at varied intervals (24, 48, 60, 72, and 96 h). Figure 4 shows the tracking of embryo development observed with an optical microscope at varied times. It indicates that there is no harm to the oocytes and sperms after p-DEP trapping and IVF in the medium of low conductivity.

● DEP microfluidic channel experiment

**Figure 5:**
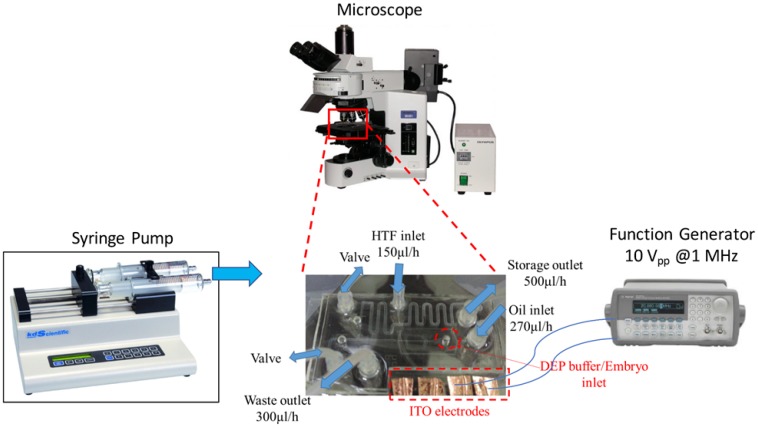
Experimental setup. The outlet connects a syringe pump to apply a steady flow field and to control the rate of volume flow. The ITO electrodes of our DEP microfluidic IVF biochip connect to the function generator to regulate the AC voltage and the frequency. The entire process was observed with an optical microscope.

The experimental settings are shown in Figure 5. The ITO pads of a DEP microfluidic IVF chip were connected to a function generator (Agilent, 5301 Stevens Creek Blvd, Santa Clara, CA 95051, USA) to regulate the AC voltage and frequency. The syringe pump (KD Scientific Syringe Pumps, Holliston, MA, USA, KDS 220) supplied a steady and laminar flow field through control of the small rate of volume flow. The experimental process was observed with an optical microscope (Olympus BX51, Olympus Corporation, Shinjuku-ku, Tokyo, Japan).

**Figure 6:**
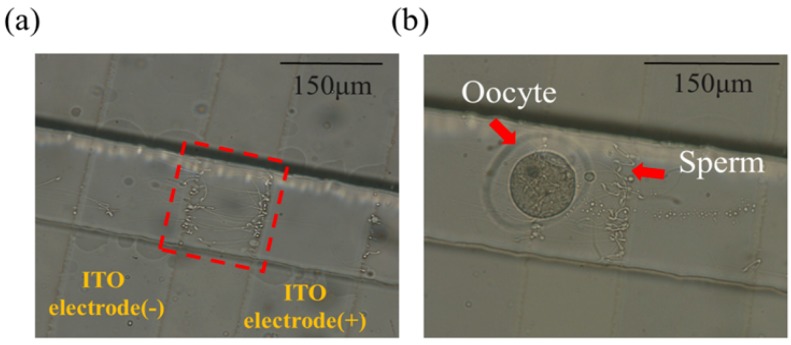
(**a**) Sperms were accumulated between the ITO electrodes by the p-DEP force. (**b**) The sperm concentration in the vicinity of an oocyte was increased.

The experiment was operated in the following steps. First, sperms at a varied sperm-oocyte ratio (500 and 2000) within a buffer solution flowed from the buffer inlet at 30 µL/h so as to be captured on DEP electrodes, as shown in Figure 6a. To manipulate the oocytes and sperms based on the p-DEP force, the condition of an AC pulse was set to 10 Vpp at a frequency of 1 MHz [27]. Furthermore, 10–15 oocytes were washed three times with an oral pipette in a DEP buffer solution to maintain the small conductivity and added into the same inlet to be co-trapped with sperms for 30 s, as shown in Figure 6b. The electric field was switched off to release the target sample that flowed into the droplet generation area. Accordingly, the microdroplets, with or without encapsulated oocytes and sperms, were sorted using pneumatic valves. The microdroplets with embryos formed by oocytes and sperms entered the storage area; the others were separated into a waste reservoir. The buffer solution used on the chip for sperms and oocytes was a DEP buffer solution in order to generate the DEP effect on the electrodes. The conductivity of the DEP buffer solution was 0.00056 S/m, which would not be suitable for further embryo culture. Thus, we transferred the sorted embryos to a Petri dish by pipette for culture in KSOM buffer solution. The human tubular fluid (HTF) medium inlet was designed for the exchange of buffer solution in further embryo culture.

## 3. Results and Discussion

**Figure 7:**
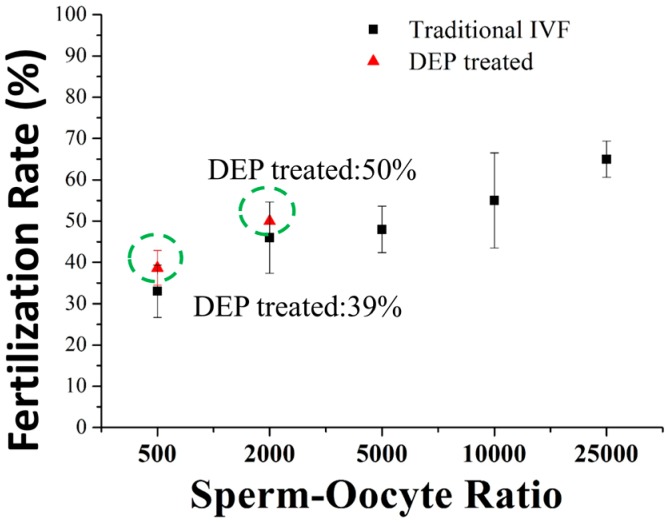
Comparison of fertilization rates with a DEP microfluidic IVF biochip and traditional IVF at varied sperm-oocyte ratios. The fertilization rate was about 5% greater with DEP treatment than with traditional IVF.

In the experiment, the number of concentrated sperms was roughly calculated and evaluated by counting under a microscope. The increased number of sperms can be easily studied before and after the applied the voltage. We observed that the local sperm concentration increased 20 to 40 times from the original sperm concentration. Figure 7 shows the rate of in vitro fertilization of a DEP microfluidic IVF chip and that of the traditional IVF process at varied sperm-oocyte ratios. Whether using our DEP microfluidic IVF chip or a standard IVF method, the fertilization rate rose in correspondence with the sperm-oocyte ratio. At a sperm-oocyte ratio of 500, the fertilization rate was 39.4% in our DEP microfluidic IVF chip. Moreover, at a sperm-oocyte ratio of 2000, the fertilization rate was 50.2%. Both fertility results were 5% greater, but not significantly greater than those achieved by the traditional IVF groups.

**Figure 8:**
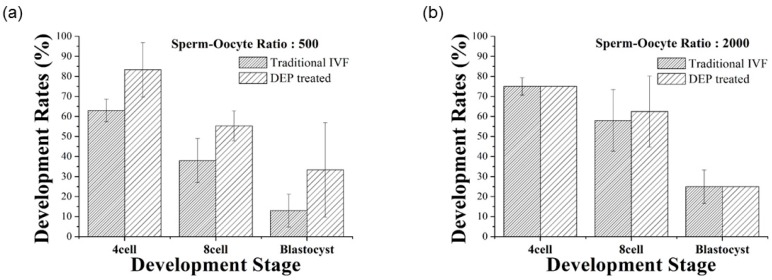
Comparison of development rates with a DEP microfluidic IVF biochip and with traditional IVF at sperm-oocyte ratios of (**a**) 500 and (**b**) 2000. The DEP-treatment experiment provided development rates that improved more than 20%, but that were not significantly improved relative to the traditional IVF group at a smaller sperm-oocyte ratio. Values are the mean ± SD of measurements from three representative experiments.

As shown in Figure 8, the blastocyst rates of the DEP microfluidic IVF chip were 33.3% and 25.1% at sperm-oocyte ratios of 500 and 2000, respectively. Furthermore, the development rate was 20% greater, but not significantly greater for the DEP microfluidic IVF experiment compared to the traditional IVF method.

The results of the present work demonstrate the achievement of a DEP microfluidic IVF biochip that enhances the rate of fertilization of oocytes in vitro with a low sperm concentration. In this research, a positive dielectrophoretic (p-DEP) force appears to trap the mouse oocytes and sperms at a specific region in the microchannel within the microfluidic chip. For this device to succeed as a usable IVF technique, the fertilized oocytes must be separated from unfertilized oocytes and subsequently cultured to the development stages of an embryo in the microfluidic chip. The result of the DEP microfluidic IVF chip shows that the sperm concentration can be increased with this trapping method. The fertilization rate was increased 5% with DEP treatment beyond that in traditional IVF, and even more than 20% developed to the blastocyst stage with a smaller sperm-oocyte ratio. Table 1 shows the compared results of fertilization rates between our study and other reports [23]. It is obvious that the fertilization rate can be increased by using the developed microfluidic system, especially for a small sperm-oocyte ratio.

Intracytoplasmic sperm injection (ICSI) is a successful treatment for severe male-factor infertility, enabling fertilization using ejaculated semen samples with extremely poor conventional sperm parameters [28,29]. In the conventional ICSI method, a fraction of the sperm suspension is added to the periphery of a microdroplet covered with mineral oil, and the sperm to be injected is selected upon inserting a microinjection pipette into the microdroplet. Sperm selection using the conventional ICSI method is a time-consuming process, particularly at sperm concentrations <10^4^ cells/mL [30].

The proportion of ART cycles involving ICSI performed in the USA increased steadily from 1996 through 2012. The use of ICSI doubled during that period, from 36.4% to 76.2% of all fresh IVF cycles, with the greatest increase occurring in cycles without male-factor infertility. Compared with conventional IVF, the use of ICSI was not associated with improved post-fertilization reproductive outcomes, regardless of the male-factor infertility diagnosis [31]. The rate of fertilization of oocytes and the rate of single sperm penetration can reflect the efficiency of IVF. Rates of fertilization and single sperm penetration are most likely to be influenced by the sperm concentration in IVF. The rate of single sperm penetration and the cleavage rate decreased significantly when the sperm concentration was at 1 × 10^6^ and 5 × 10^6^ sperm/mL with a microfluidic sperm sorter [32], demonstrating that the cleavage and blastocyst rates did not decrease significantly when the sperm-egg ratio decreased from 1200:1 to 600:1. However, when the sperm-egg ratio was 300:1, the cleavage and blastocyst rates decreased significantly [33].

A possible reason to replace ICSI treatment with the use our device is to enhance the slight increase in sperm concentration in the microfluidic channels. We designed the structure of the ITO-glass electrode chip and microfluidic channels to keep the sperms and oocytes at the bottom of the chip using a positive dielectrophoretic (p-DEP) force at the ITO-glass electrode. In addition, a flow-focusing microfluidic device made of polydimethylsiloxane (PDMS) was used to generate microdroplets to contain the zygotes. The volume of microdroplets can be controlled by adjusting the flow rates of both inlets for oil and the DEP buffer. These results indicate that the increases did not depend on the concentration of sperms in the loaded semen; a significant difference in the sperm-oocyte ratio was thus observed at 500 loaded. A positive DEP force served to drive the position of the oocyte and the sperms to facilitate natural fertilization in our microchannel structures at an insemination concentration.

## 4. Conclusions

We have demonstrated a DEP microfluidic IVF biochip that is able to enhance the rate of in vitro fertilization. In this research, a voltage (10 Vpp and 1-MHz sine wave) was applied to generate a strong electric field; a positive dielectrophoretic (p-DEP) force appeared to trap the mouse oocytes and sperms at a specific region in the microchannel. The result of the DEP microfluidic IVF chip shows that the sperm concentration was increased with this trapping method. In this research, the fertilization rate was increased by 5% with DEP treatment, in comparison to the traditional IVF method, and even more than 20% developed to the blastocyst stage with a smaller sperm-oocyte ratio. Our future work will involve developing a microfluidic chip for techniques of in vitro assisted reproduction in the infertility therapy field.

## Figures and Tables

**Table 1:** Comparison of fertilization rate achieved in our study and other reports.

Sperm-Oocyte Ratio	Fertilization Rate [23]	Fertilization Rate by Traditional IVF	Fertilization Rate by Microfluidic System
200	26.3%		
400/500	27.1%	33–38%	~39%
750	18.0%		
2500/2000	8.1%	44–56%	~50%
5000	12.0%	45–53%	~53%
10,000	N/A*	53–62%	~58%
25,000	N/A	66–74%	~65%

*N/A, not applicable.
